# Is PONV still a problem in pediatric surgery: a prospective study of what children tell us

**DOI:** 10.3389/fped.2023.1241304

**Published:** 2023-10-30

**Authors:** Brigitte Messerer, Marko Stijic, Andreas Sandner-Kiesling, Johanna M. Brillinger, Jasmin Helm, Jacqueline Scheer, Christof Stefan Strohmeier, Alexander Avian

**Affiliations:** ^1^ Department of Anesthesiology and Intensive Care Medicine, Medical University of Graz, Graz, Austria; ^2^ Institute for Medical Informatics, Statistics and Documentation, Medical University of Graz, Graz, Austria; ^3^ University Clinic for Neurology, Clinical Department for Neurogeriatrics, Medical University of Graz, Graz, Austria

**Keywords:** pediatrics, postoperative nausea and vomiting, prevalence, risk factors, analgesics, antiemetic

## Abstract

**Background:**

Postoperative nausea and vomiting (PONV) is an unpleasant complication after surgery that commonly co-occurs with pain. Considering the high prevalence among pediatric patients, it is important to explore the main risk factors leading to PONV in order to optimize treatment strategies. The objectives of this study are as follows: (1) to determine the prevalence of PONV on the day of surgery by conducting interviews with pediatric patients, (2) to assess PONV prevalence in the recovery room and on the ward by analyzing nursing records, and (3) to collect information on PONV risk factors on the day of surgery and the following postoperative days. We wanted to investigate real-life scenarios rather than relying on artificially designed studies.

**Methods:**

A prospective analysis [according to STrengthening the Reporting of OBservational studies in Epidemiology (STROBE) guidelines] of PONV on the day of surgery and the following postoperative days was conducted by evaluating demographic and procedural parameters, as well as conducting interviews with the children under study. A total of 626 children and adolescents, ranging in age from 4 to 18 years, were interviewed on the ward following their surgery. The interviews were conducted using a questionnaire, as children aged 4 and above can participate in an outcome-based survey.

**Results:**

On the day of surgery, several multivariable independent predictors were identified for PONV. The type of surgery was found to be a significant factor (*p* = 0.040) with the highest odds ratio (OR) in patients with procedural investigations [OR 5.9, 95% confidence interval (CI): 1.8–19.2], followed by abdominal surgery (OR 3.1, 95% CI: 0.9–11.1) when inguinal surgery was used as the reference category. In addition, the study identified several predictors, including the amount of fentanyl administered during anesthesia (µg/kg body weight) (OR 1.4, 95% CI: 1.1–1.8), intraoperative use of piritramide (OR 2.6, 95% CI: 1.5–4.4) and diclofenac (OR 2.0, 95% CI: 1. 3–3.1), opioid administration in the recovery room (OR 3.0, 95% CI: 1.9–4.7), and piritramide use on the ward (OR 4.5, 95% CI: 1.7–11.6).

**Conclusions:**

The main risk factors for PONV include the intraoperative administration of opioids during the recovery room stay and at the ward, the intraoperative use of non-opioids (diclofenac), and the specific type of surgical procedure. Real-life data demonstrated that in clinical praxis, there is a gap between the adherence to established guidelines and the use of antiemetic prophylaxis in surgeries that are generally not associated with a high PONV prevalence. Further efforts are needed to improve the existing procedures and thus improve the overall outcome.

## 1. Introduction

Postoperative nausea and vomiting (PONV) is, along with pain, an unpleasant complication that can occur in both adults and children following surgical interventions and administration of anesthesia (1, 2). In addition to the strong impairment of the subjective state of mind, PONV has been found to diminish child and parental satisfaction and can cause statistically significant resource use, including prolonged recovery time and unplanned hospitalization (3). Moreover, serious medical complications may occur, such as aspiration, dehydration, postoperative bleeding, airway obstruction, intracranial pressure elevation, suture dehiscence, or electrolyte imbalance (4).

The prevalence rate of PONV in children is alarmingly high, reported to range from 30% in the general surgical population to 80% in high-risk pediatric postoperative patients (3, 5–8). Thus, risk assessment and prophylaxis strategies to avoid or to reduce PONV in children have gained considerable attention (3, 9).

The risk factors vary between adults and children (3, 7, 8, 10, 11). Hence, the risk scores validated in adults are not applicable in children (5). Anesthetic factors have been found to influence the prevalence of PONV in pediatric patients such as the performance of general anesthesia (7, 12), application of opioids and their increased use (9), administration of volatile anesthetics (13), use of nitrous oxide (3) and anticholinesterases, and less use of perioperative fluids (14). In addition, there are surgical factors that also influence the prevalence of PONV such as the duration of surgery longer than 30 min (7, 12), ocular surgeries (strabismus correction), ENT surgeries (adenoidectomy, tonsillectomy) (15), strabismus surgery (16), otoplasty, laparoscopic surgery, orchidopexy, and hernia repair (17). Known individual factors that influence the prevalence of PONV include age ≥3 years (peak between 6 and 10 years) (18), post-pubertal females (3), pain (9), known former PONV episodes in the child or their immediate relatives (parents or siblings), motion sickness, prolonged preoperative fasting, and dehydration. It is quite possible that efforts to assess and reduce the risk factors will lead to decreased PONV prevalence (3).

The aim of this study is to determine PONV prevalence in children and adolescents as an indicator of the quality of our local intraoperative and postoperative standard procedures (antiemetic prophylaxis, use of medications, and regional anesthesiologic procedures) from the children's perspective, to identify deficiencies and to learn more about potential risk factors to improve patient outcomes. At the age of 4–6 years, children can self-assess pain intensity (19) as they can distinguish between “more,” “less,” or “the same” at this age and usually complete an outcome-based questionnaire with support (20).

The aim of this study is to determine PONV prevalence in our pediatric recovery room and postoperatively on the ward. In addition, we aim to define possible risk factors for the occurrence of PONV. For this purpose, we interviewed children on the ward on the day of surgery and recorded demographic parameters as well as entries in the patient chart.

## 2. Methods

### 2.1. Sample

We conducted this cross-sectional study over a 9-month period (from 12 February 2018 to 21 March 2018 and between 11 October 2018 and 29 May 2019) at the Department of Pediatric and Adolescent Surgery of the Medical University Hospital of Graz, Austria. The study was conducted in accordance with the Declaration of Helsinki 1996, Good Clinical Practice, and applicable local regulatory requirements and law. The target population were children and adolescents, aged 4–18 years (*N* = 626), who were cared by pediatric anesthesiologists in the operating rooms and received postoperative care in the recovery room of the Department of the Pediatric and Adolescent Surgery. Only patients with prior written parental consent for children up to 14 years and child and parental consent for children over 14 years were included. This manuscript is written according to the STrengthening the Reporting of OBservational studies in Epidemiology (STROBE) criteria in its current version (21). Surgeries performed in other departments were excluded from the study (ear, nose, and eye surgeries). In addition, patients with insufficient German language skills, patients below the age of 4 (younger children are not able to participate in an outcome-based survey) and beyond the age of 18 (from the age of 18, treatment takes place in adult departments), patients with cognitive impairment, patients who are receiving intensive care, patients who refuse to participate, and patients whose general condition prevents them from being interviewed were also excluded from the study (20).

The local ethics committee of the Medical University of Graz, Austria, approved the study protocol (EC number 29-262 ex 16/17).

### 2.2. Procedure

A team of pediatric surgeons and 12 pediatric anesthetists provided care for pediatric patients at the Department of Pediatric and Adolescent Surgery at the Medical University Hospital in Graz, Austria. Two to three young colleagues were in rotation, but were always accompanied by a pediatric anesthetist. Only a few surgeons from other departments (dentists, oral surgeons, neurosurgeons) came to the Children's Center to perform their operations on children, but they too specialized in children.

The intra- and postoperative procedure was standardized. For typical and frequent surgeries in children (e.g., circumcisions, herniotomies, appendectomies, fracture care, pediatric orthopedic operations), intervention-specific recommendations according to best clinical practice, scientific principles, and guidelines were available at our institution (22, 23). To reduce the prevalence of PONV, nitrous oxide was not used and volatile anesthetics were avoided wherever feasible, in favor of employing total intravenous anesthesia with propofol. The focus was on ensuring adequate hydration and assessment of risk factors for PONV. In the event that any were present, our standard procedures included the use of dexamethasone (0.1 mg kg^−1^, maximum 4 mg, at the beginning of surgery) or ondansetron (0.1 mg kg^−1^, maximum dose 4 mg, at the end of surgery) as a pharmacological prophylaxis strategy.

Another cornerstone of PONV prophylaxis is a multimodal pain management to reduce intra- and postoperative opioid requirements (1). Therefore, we administered non-opioid medications immediately following the induction process, and scheduled them regularly in the patient's chart, as these medications are often sufficient to treat low to moderate pain. The opioids administered during the intraoperative period were fentanyl, piritramide, or remifentanil. We performed a local/regional anesthetic technique whenever possible (24). When a patient experienced intense and prolonged postoperative pain, the optimal choice would be to administer patient-controlled regional analgesia via catheter.

In the recovery room, we treated a pain score of ≤3 with non-opioids and treated moderate to severe pain (VAS ≥4) with opioids. In cases of highly scored pain, which lasted for a longer postoperative period, an intravenous patient-controlled analgesia was used, if regional analgesia due to type of surgery, patient/parents refusal or contraindication (e.g., coagulation disorders, severe infections at the site of catheter insertion, generalized sepsis, allergy to local anesthetics, neuromuscular disease) could not be performed (25).

Oral intake was allowed and required after full recovery, unless there was a surgical contraindication. We assumed that children who were not well would not want to drink (26). If a child vomited and/or was severely nauseated, ondansetron was the option. A patient was discharged from the recovery room in accordance with standard clinical practice: awake, no agitation, hemodynamic stability, clear airway, no apparent bleeding, no nausea or vomiting, and a maximum pain level of ≤2.

On the ward, patients were administered non-opioids prophylactically on a regular basis during the first postoperative days, while opioids (primarily piritramide) were prescribed for the treatment of breakthrough pain. The duration of patient-controlled analgesia was determined based on the patient’s pain values and requirements. Ondansetron was the first line antiemetic for postoperative vomiting (POV)/PONV treatment on demand on the ward.

In our department, there was a standardized written procedure for the regular assessment of pain and complaints such as nausea and vomiting. Upon admission to the hospital, the nursing staff informed both children and parents that pain and complaints such as nausea/vomiting were evaluated regularly during each care visit (in the morning, at noon, in the evening, and at night). In addition, the children/parents were instructed to report spontaneous occurrences. At our facility, we assessed pain in a standardized way until discharge using Hicks’ revised Faces Pain Scale (FPS-r) (19) for children aged 4 years and older when self-assessment was possible. Due to the non-linear character of children's pain ratings (27), the pain scores were categorized for analysis. In addition to pain assessment, side effects were also checked and noted. If needed, the prescribed analgesic on demand or the ordered antiemetic was administered. After 30 min, a reassessment was conducted to check for effectiveness (28). If the intervention was ineffective, the doctor was called to rule out a possible complication. The nursing stuff recorded all applications, side effects (incidents of nausea and vomiting), evaluated pain values, and physiological indexes in the patient's chart.

### 2.3. Material

To assess the prevalence of PONV and other important factors (e.g., pain) in an outcome-oriented way, we developed a questionnaire. The questionnaire was given to the patient and filled out by themselves without any help, with help (having the questions read aloud or explained), or someone else (e.g., parents) did it for them. The way it was answered was also recorded. PONV was detected by the following: (1) the item about nausea or vomiting within the questionnaire that could be answered with “yes,” “no,” or “do not know”; (2) episodes of nausea or vomiting that were noted and documented by nurses in the recovery room and on the ward; and (3) application of ondansetron in the recovery room or on the ward. For the pain evaluation, we used the FPS-r according to Hicks et al. (19), as already mentioned. We assessed pain on the surgical side and abdominal pain separately.

After full recovery on the ward, an independent researcher, not involved in patient care, provided the questionnaire to the patients who had given their written consent to participate in the study.

We looked at demographic and procedural parameters such as gender, age, weight, type of surgery, duration of surgery and anesthesia, type of anesthesia used, the drugs used, and the analgesics and antiemetic administered in the recovery room and ward.

### 2.4. Statistical analysis

We have presented continuous data as medians (min–max) and categorical data as proportions. In the case of children and adolescents, it is important to account for the specific age and sex of the patient when interpreting body mass index (BMI) scores, as “normal” BMI values vary with age. To address this, the BMI *z*-score was used instead of the BMI value. The BMI *z*-score was calculated according to WHO Children Growth Standards (29). The BMI *z*-score indicates how many standard deviations a child deviates from the average BMI of the corresponding age and gender. A BMI *z*-score of “0” refers to the expected body mass index of this age group and sex. A BMI z-score of “−1” corresponds to a body mass index score that is one standard deviation beneath the expected body mass index score for this age and sex, and a BMI *z*-score of “+1” corresponds to a body mass index score that is one standard deviation above the expected body mass index score for this age and sex.

Logistic regression was employed to construct models that established the relationship between the occurrence of nausea, vomiting, and both nausea and vomiting in patients and independent predictors. In the first step, univariable logistic regression analyses were performed. This study analyzed the impact of many factors, including age, sex, BMI *z*-score, medication, type of surgery, and duration of surgery, on the frequency of occurrence of nausea, vomiting, and both nausea and vomiting throughout the operation day as well as the following postoperative days. The odds ratios (ORs) and 95% confidence intervals (CIs) were calculated.

To avoid the problems of multicollinearity between individual risk factors, a formal detection-tolerance or the variance inflation factor (VIF) for multicollinearity has been applied. According to O’Brian (30), a tolerance value of less than 0.20 or 0.10, or a VIF of 5 or 10 and above, indicates a multicollinearity problem. Factors that were statistically significant in univariable analysis and that showed no multicollinearity were submitted to logistic multivariable regression analysis (forward stepwise method). Multivariable analyses were conducted if there were at least 20 cases in the univariable analyses and if their univariable *p*-values were <0.200. Missing data were not imputed.

All statistical analyses were performed using the Statistical Package for Social Sciences version 25.0 (SPSS Inc., Chicago, IL, USA) or SAS 9.2 (SAS Institute Inc., Cary, NC, USA). A two-sided *p* < .05 was considered to be statistically significant.

## 3. Results

### 3.1. Descriptive statistics

Between February 2018 and May 2019, a cohort of 626 pediatric patients (aged 4–17 years) who had undergone surgery at the Children's Department of the University clinic in Graz were included in this study. A total of 405 patients were male (64.7%). The median age of our patients was 11.1 years (the youngest child was 4 years, and the oldest adolescent was 18). The median body mass index *z*-score was 0.32 (range: 4.9–5.1; female: 0.46 [3.7–5.1); male: 0.26 (4.9–4.3), *p* = 0.328]. A total of 332 patients (53.0%) received general anesthesia, 278 patients (44.4%) received general anesthesia plus regional anesthesia, and 15 patients (2.4%) received only sedoanalgesia. Only one patient (0.2%) had spinal anesthesia. A total number of 615 children (98.2%) got opioids during the surgery, 213 patients (34.0%) in the recovery room, and just 25 children (4%) on demand on the day of surgery on the ward (Table 1 and Supplementary Material Table S1).

**Table 1:** Patient characteristics (*n* = 626).

Variables	*n* (%) median (min–max)	PONV prevalence on the day of surgery (including recovery room)
Sex		
Male	405 (64.7)	19.5% (*n* = 79)
Female	221 (35.3)	26.2% (*n* = 58)
Age in years	11.08 (4.04–17.99)	
Age groups (years)		
4 ≤ 6	70 (11.2)	17.1% (*n* = 12)
6 ≤ 10	200 (31.9)	24.0% (*n* = 48)
10 ≤ 13	128 (20.4)	22.7% (*n* = 29)
13 ≤ 18	228 (36.4)	21.1% (*n* = 48)
Body mass index *z*-score	.32 (−4.92–5.09)	
Body mass index *z*-score^a^		
≥1	103 (16.5)	17.5% (*n* = 18)
−1 to 1	319 (51.0)	22.6% (*n* = 72)
>1	202 (32.3)	23.3% (*n* = 47)
ASA score		
1	455 (72.7)	21.5% (*n* = 98)
2	165 (26.4)	22.4% (*n* = 37)
3	6 (1.0)	(*n* = 2)
Duration of surgery in min	31 (1–348)	
Duration of anesthesia in min	68 (9–434)	
Duration of anesthesia − duration of surgery in min	36 (4–40)	
History of PONV/motion sickness		
No	589 (94.1)	21.9% (*n* = 129)
PONV	33 (5.3)	24.2% (*n* = 8)
Motion sickness	4 (0.6)	(*n* = 0)
Type of surgery		
Inguinal surgery	48 (7.7)	14.6% (*n* = 7)
Plastic surgery	27 (4.3)	18.5% (*n* = 5)
Bones	189 (30.2)	24.3% (*n* = 46)
Knee, shoulder, hip, joints	55 (8.8)	23.6% (*n* = 13)
Minor surgery	129 (20.6)	20.9% (*n* = 27)
Urogenital tract	113 (18.1)	15.0% (*n* = 17)
Abdominal procedures	23 (3.7)	52.2% (*n* = 12)
Investigations^b^	42 (6.7)	23.8% (*n* = 10)
Recovery room: time of stay in min	104 (19–276)	** **
Maximal pain—recovery room		
0–3 FPS-r	452 (72.2)	16.2% (*n* = 73)
≥4 FPS-r	174 (27.8)	36.8% (*n* = 64)
Pain: day of surgery on the ward		
0 FPS-r	330 (52.8)	18.2% (*n* = 60)
1–3 FPS-r	215 (34.4)	21.4% (*n* = 46)
4–10 FPS-r	80 (12.8)	38.8% (*n* = 31)
Pain: first postoperative day		
0 FPS-r	138 (43.8)	
1–3 FPS-r	103 (32.7)	
4–10 FPS-r	74 (23.5)	
Pain: second postoperative day		
0 FPS-r	95 (49.2)	
1–3 FPS-r	65 (33.7)	
4–10 FPS-r	33 (17.1)	
Pain: maximal pain level during hospital stay on the ward		
0 FPS-r	295 (47.1)	
1–3 FPS-r	202 (32.3)	
4–10 FPS-r	129 (20.6)	
PONV: day of surgery (recovery room + ward)		
No	489 (78.1)	
Yes	137 (21.9)	
PONV: first postoperative day		
No	303 (96.2)	
Yes	12 (3.8)	
PONV: second postoperative day		
No	185 (96.4)	
Yes	7 (3.6)	

^a^ Although we knew the height and weight, no calculation for the body mass index z-score was possible for two children who were too young.

^b^ Investigations: gastroscopy *n* = 26, rectoscopy *n* = 7, colonoscopy *n* = 6, cystoscopy *n* = 2, and bronchoscopy *n* = 1.

Propofol was used for induction (99.4%) as well as maintenance (98.1%) of anesthesia. Non-opioids were administered intraoperatively in 82.1% (*n* = 519) of the cases, with non-steroidal anti-inflammatory drugs (NSAIDs) being the preferred non-opioid option for 479 patients. In the recovery room, we applied a non-opioid to 175 patients (28%), with metamizole being the most commonly used (*n* = 167). Co-analgesics, including ketamine (16.3%) and clonidine (18.5%), were administered intraoperatively in 31.5% of children. Clonidine (*n* = 104) played the largest role among the co-analgesics (*n* = 105) used in the recovery room. We performed regional anesthesia in 282 (45%) patients. The most common procedure was caudal epidural anesthesia (*n* = 82; 12.9%), followed by local infiltration and penile nerve block (*n* = 48; 7.7%). Regional catheters were inserted in 28 children (*n* = 2 epidural; *n* = 26 peripheral) (Table 2). A PONV prophylaxis in the form of intraoperative antiemetic administration was performed in 133 patients. Dexamethasone, ondansetron, and droperidol were administered at 15.5%, 8.5%, and 2.1% of the cases, respectively.

**Table 2:** Intraoperative medication, medication at the recovery room and at the ward.

Intraoperative	*n* (%) or median (min–max)
Induction of anesthesia	
Propofol	622 (99.4)
Other	4 (0.6)
Maintenance of anesthesia	
Propofol	614 (98.1)
Other	12 (1.9)
Opioids (intraoperative)	
No	11 (1.8)
Yes	615 (98.2)
Piritramide	
No	284 (45.4)
Yes	342 (54.6)
Piritramide, µg/kg BW	79.6 (0.0–374.3)
Fentanyl	
No	60 (9.2)
Yes	566 (90.4)
Fentanyl, µg/kg BW	2.00 (0.00–8.57)
Remifentanil	
No	375 (59.9)
Yes	251 (40.1)
Remifentanil, µg/kg BW	0.00 (0.00–55.43)
Non-opioid (intraoperative)	
No	107 (17.1)
Yes	519 (82.1)
Ibuprofen	
No	526 (84.0)
Yes	100 (16.0)
Ibuprofen, mg/kg BW	0.00 (0.00–12.5)
Diclofenac	
No	422 (67.4)
Yes	204 (32.6)
Diclofenac, mg/kg BW	0.00 (0.00–2.27)
Neodolpasse® (diclofenac + orphenadrine)	
No	451 (72.0)
Yes	175 (28.0)
Neodolpasse®, ml/kg BW	0.00 (0.00–4.39)
Metamizole	
No	581 (92.8)
Yes	45 (7.2)
Metamizole, mg/kg BW	0.00 (0.00–15.79)
Co-analgesics (intraoperative)	
No	429 (68.5)
Yes	197 (31.5)
S-ketamine	
No	524 (83.7)
Yes	102 (16.3)
S-ketamine, mg/kg BW	0.00 (0.00–2.34)
Clonidine	
No	510 (81.5)
Yes	116 (18.5)
Clonidine, µg/kg BW	0.00 (0.00–4.44)
Antiemetic prophylaxis	
No	493 (78.8)
Yes	133 (21.2)
Dexamethasone—intraoperative	
No	529 (84.5)
Yes	97 (15.5)
Dexamethasone—intraoperative, mg/kg BW	0.00 (0.00–1.09)
Droperidol—intraoperative	
No	613 (97.9)
Yes	13 (2.1)
Ondansetron—intraoperative	
No	567 (91.5)
Yes	53 (8.5)
Ondansetron—intraoperative, mg/kg BW	0.00 (0.00–0.13)
Regional anesthesia	
No	344 (55.0)
Yes	282 (45.0)
Catheter	
No	598 (95.5)
Yes	28 (4.5)
Recovery room	
Opioids	
No	413 (66.0)
Yes	213 (34.0)
Piritramide	
No	442 (70.6)
Yes	184 (29.4)
Piritramide, µg/kg BW	0.0 (0.0–153.4)
Nalbuphine	
No	606 (96.8)
Yes	20 (3.2)
PCA i.v.	
No	612 (97.8)
Yes	14 (2.2)
Non-opioid	
No	451 (72.0)
Yes	175 (28.0)
Metamizole	
No	459 (73.3)
Yes	167 (26.7)
Metamizole, mg/kg BW	0.00 (0.00–20.00)
Neodolpasse® (diclofenac + orphenadrine)	
No	619 (98.9)
Yes	7 (1.1)
Co-analgesics	
No	521 (83.2)
Yes	105 (16.8)
Clonidine	
No	522 (83.4)
Yes	104 (16.6)
Clonidine, µg/kg BW	0.00 (0.00–2.88)
S-ketamine	
No	618 (98.7)
Yes	8 (1.3)
Antiemetic	
Ondansetron	
No	614 (98.1)
Yes	12 (1.9)
Total amount of Piritramide intraop. + recovery room, µg/kg BW	91.8 (0.0–374.3)
On the ward—on the day of surgery	
Opioids	
Piritramide	
No	601 (96)
Yes	25 (4.0)
PCA i.v.	
No	612 (97.8)
Yes	14 (2.2)
PCA regional	
No	598 (95.5)
Yes	28 (4.5)
Non-opioid	
No	342 (54.6)
Yes	284 (45.4)
Ondansetron	
No	548 (87.5)
Yes	78 (12.5)

A total of 189 children (31.1%) reported a maximum pain score of ≥4 in the recovery room, while 423 (67.6%) patients were transferred back to the ward pain-free and 23 patients were transferred although they did not meet the discharge criteria with a pain score of 3 and 4, respectively. On the day of surgery, 52.8% (*n* = 330) of our children on the ward experienced no pain, and only 80 children (12.8%) reported a pain score of ≥4). To the question, “Would you have liked to receive more pain medication?” 88.6% answered no and 11.4% replied yes (Table 3).

**Table 3:** Self-reported pain and PONV in the recovery room, documented pain and PONV at the recovery room or ward.

Recovery room—self-reported (questionnaire administered 1–2 h after transfer to the ward)	*n* (%)
How severe was your pain at the site of the surgery?	
0	244 (40.9)
1–3	189 (31.7)
≥4	163 (27.3)
Did you have pain outside of the surgical area?	
How severe was it?	
0	499 (83.9)
1–3	55 (9.1)
≥4	41 (7.0)
Where did it hurt?	
Abdominal pain	42 (6.7)
Headache	34 (5.4)
Muscle pain	18 (2.9)
Shoulder pain	16 (2.6)
Back pain	6 (1.0)
Abdominal pain	42 (6.7)
Pain in another place	127 (20.3)
Maximal pain reported	
0	197 (32.5)
1–3	221 (36.4)
≥4	189 (31.1)
Would you have liked to receive more pain medication? *n* = 561	
No	497 (88.6)
Yes	64 (11.4)
I don't know	53 (8.5)
Missing	12 (1.9)
Were you nauseous?	
No	543 (90.7)
Yes	56 (9.3)
I don't know	15 (2.4)
Missing	12 (1.9)
Have you vomited?	
No	583 (95.3)
Yes	29 (4.7)
I don't know	7 (1.1)
Missing	7 (1.1)
Were you tired?	
No	71 (11.3)
Yes	545 (87.1)
I don't know	8 (1.3)
Missing	2 (0.3)
Dry mouth	
No	187 (29.9)
Yes	385 (61.5)
Missing	54 (8.6)
Wish to receive more pain medication?	
No	497 (79.4)
Yes	64 (10.2)
Missing	65 (10.4)
How did you answer the questions?	
All alone	192 (30.7)
With help (read out, explain)	321 (51.3)
Someone else (e.g. parents) did it for me	96 (15.3)
Missing	17 (2.7)
Recovery room—documented	
Pain value when transferred to the inpatient	
≤2	603 (96.3)
3/4	23 (3.7)
On the ward—on the day of surgery—documented	
Highest documented pain value	
0	330 (52.8)
1–3	215 (34.4)
≥4	80 (12.8)
Vomiting	87 (13.9)
Nausea	27 (4.3)

While in the recovery room, PONV was detected in only 6.2% (*n* = 39) cases who received ondansetron for treatment (*n* = 12, 1.9%). Specifically, nausea was documented in eight cases (1.3%), while vomiting was documented in 25 cases (4.0%). In addition, 10.2% (*n* = 63) of the patients stated nausea during the recovery room stay in the questionnaire, while 4.7% (*n* = 29) of the patients stated vomiting (Table 4). Furthermore, it was observed that 27 patients (4.3%) experienced nausea, while 87 (13.9%) patients reported vomiting on the day of surgery on the ward. The overall recorded prevalence rate of PONV was 21.9% (95% CI: 18.8–25.1) on the day of surgery, 3.8% (95% CI: 1.9–6.0) on the first postoperative day, and 3.6% (95% CI: 1.1–6.3) on the second postoperative day. The most commonly administered antiemetic on the ward was ondansetron, which was administered in 78 patients.

**Table 4:** Discrepancy between self-reported PONV and documented PONV during the recovery room stay (n = 626).

		PONV recovery room—questionnaire		
		No	Yes	
PONV recovery room—documented	No	538 (85.9%)	50 (8.0%)	588 (93.9%)
	Yes	25 (4.0%)	13 (2.1%)	38 (6.1%)
Gesamt		563 (89.9%)	63 (10.1%)	626 (100.0%)

### 3.2. Factors that predict PONV

**Figure 1:**
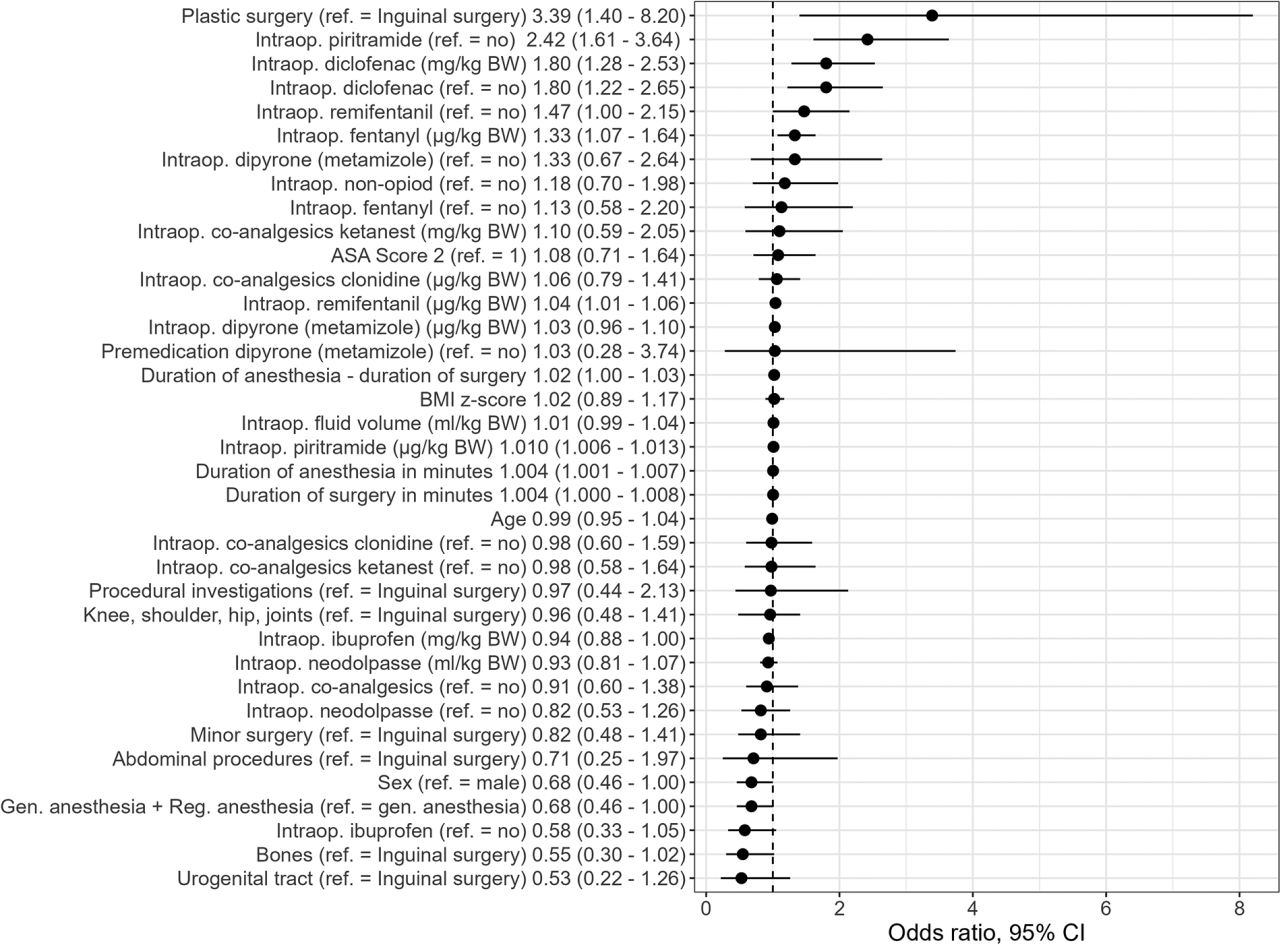
Univariate PONV predictors (sociodemographics and intraoperative factors) [odds ratios and 95% CI]. Intraop, intraoperative administration; BW, body weight.

**Figure 2:**
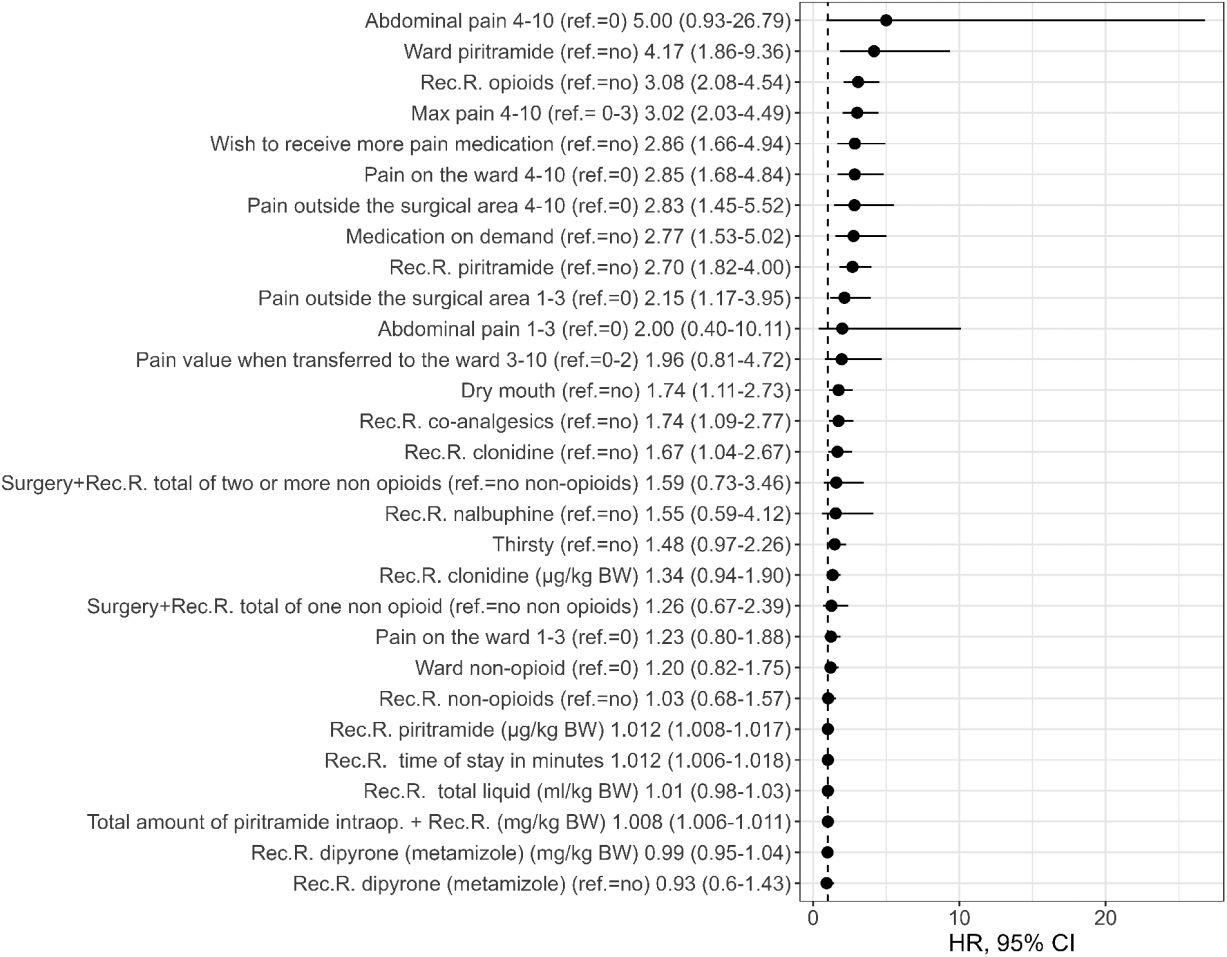
Univariate PONV predictors (on ward) [odds ratios and 95% CI]. Intraop, intraoperative administration; Rec.R., recovery room; BW, body weight.

The intraoperative variables that were found to be univariable statistically significant in predicting PONV on the day of surgery included the type of surgery [with the highest OR in patients with abdominal procedures (OR 6.38, 95% CI: 2.03–20.0), when inguinal surgery was used as the reference category], duration of surgery, duration of anesthesia, and intraoperative application of piritramide, NSAID (diclofenac, *p* = .003), remifentanil, and fentanyl (see Figure 1 and Supplementary Material Table S2). In the recovery room, the length of stay, maximum pain score, dry mouth, pain outside the surgical area, desire for more analgesics, and administration of opioids (particularly piritramide) and co-analgesics (particularly the use of clonidine) were statistically significant univariable predictors of PONV. A pain score of ≥4 and thus the administration of an on-demand medication, particularly piritramide, were univariable predictors on the ward (see Figure 2 and Supplementary Material Table S2).

**Figure 3:**
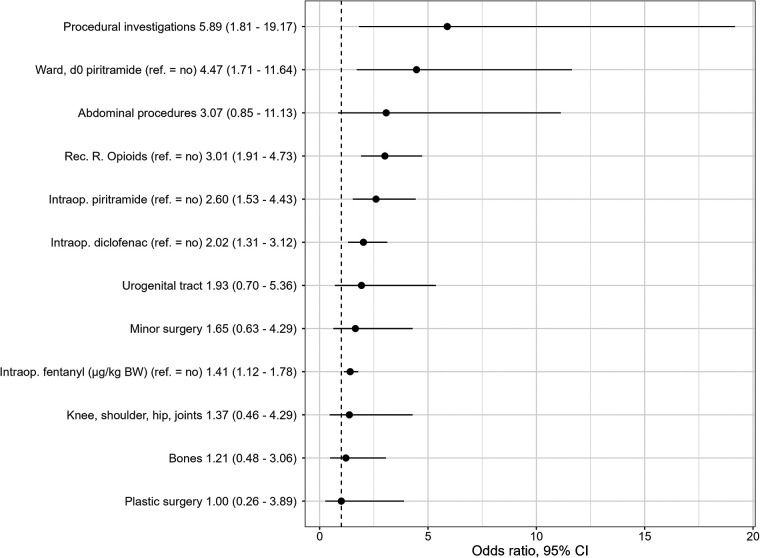
Independent statistically significant PONV predictors retained in final multivariable logistic regression model (odds ratios and 95% CI). Reference group for all surgeries, inguinal surgery; d0, day of surgery; Rec.R., recovery room; intraop., intraoperative; BW, body weight.

On the day of surgery, the multivariable independent predictors of PONV were the type of surgery (*p* = .040) with the highest OR in patients with procedural investigations (OR 5.9, 95% CI: 1.8–19.2), followed by abdominal surgery (OR 3.1, 95% CI: 0.9–11.1) and urogenital tract surgery (OR 1.9, 95% CI: 0.7–5.4) when inguinal surgery was used as the reference category. In addition, the amount of fentanyl (µg/kg body weight) (OR 1.4, 95% CI: 1.1–1.8) during anesthesia and the intraoperative use of piritramide (OR 2.6, 95% CI: 1.5–4.4) and diclofenac (OR 2.0, 95% CI: 1.3–3.1) were statistically significant predictors for PONV. The use of opioids in the recovery room (OR 3.0, 95% CI: 1.9–4.7) and the administration of piritramide on the ward (OR 4.5, 95% CI: 1.7–11.6) were also statistically significant factors for PONV (Table 5, Figure 3).

**Table 5:** Multivariate statistically significant predictors of PONV on the day of surgery (including recovery room).

Variables	*n* (%) median (min–max)	PONV prevalence	*p*-value	OR (95% CI)
Type of surgery			0.040	
Inguinal surgery	48 (7.7)	14.6% (*n* = 7)	Ref	Ref.
Plastic surgery	27 (4.3)	18.5% (*n* = 5)	1.00	1.00 (0.26–3.89)
Bones	189 (30.2)	24.3% (*n* = 46)	0.694	1.21 (0.48–3.06)
Knee, shoulder, hip, joints	55 (8.8)	23.6% (*n* = 13)	0.571	1.37 (0.46–4.29)
Minor surgery	129 (20.6)	20.9% (*n* = 27)	0.307	1.65 (0.63–4.29)
Urogenital tract	113 (18.1)	15.0% (*n* = 17)	0.207	1.93 (0.70–5.36)
Abdominal procedures	23 (3.7)	52.2% (*n* = 12)	0.087	3.07 (0.85–11.13)
Procedural investigations	42 (6.7)	23.8% (*n* = 10)	**0.003**	5.89 (1.81–19.17)
Intraoperative piritramide			**<0**.**001**	
No	284 (45.4)	14.1% (*n* = 40)	** **	Ref.
Yes	342 (54.6)	28.4% (*n* = 97)	** **	2.60 (1.53–4.43)
Intraoperative fentanyl µg/kg body weight	2.00 (0.00–8.57)		**0**.**004**	1.41 (1.12–1.78)
Intraoperative diclofenac			**0**.**002**	
No	422 (67.4)	18.5% (*n* = 78)		Ref.
Yes	204 (32.6)	28.9% (*n* = 59)		2.02 (1.31–3.12)
Recovery room: opioids			**<0**.**001**	
No	413 (66.0)	15.0% (*n* = 62)	** **	Ref.
Yes	213 (34.0)	35.2% (*n* = 75)	** **	3.01 (1.91–4.73)
Ward day of surgery: piritramide			**0**.**002**	
No	601 (96)			Ref.
Yes	25 (4.0)			4.47 (1.71–11.64)

Significant *p*-values are given bold.

## 4. Discussion

In our study, we found a PONV prevalence rate of 22% on the day of surgery, which decreased to <4% on the first and second postoperative day. The main risk factors for PONV include perioperative administration of opioids, intraoperative administration of diclofenac, and type of surgery. The observed PONV prevalence in this study was lower compared with the prevalence of the previous studies, where it ranged from 19.5% for vomiting to approximately 50% for nausea and even up to 80% in a subgroup of high-risk patients (3, 5, 14, 18). Ear–nose–throat surgery (31) and strabismus surgery (14, 32) are associated with the highest PONV prevalence (12, 33, 34), when antiemetic prophylactics are not administered. However, these types of surgeries were excluded in our study, since they were performed in other departments. This could provide some insight for the lower PONV prevalence. We have to note that a significant discrepancy exists between the documented prevalence of PONV and the events reported by the children when specifically asked regarding their experiences of nausea or vomiting during the postoperative period. In the recovery room, we recorded a prevalence rate of 1.3% (*n* = 12) for PONV. On the ward, we observed a prevalence rate of 16.1% (*n* = 101) for PONV, 13.9% (*n* = 87) for vomiting, and 4.3% (*n* = 27) for nausea on the day of surgery. Our children reported a prevalence rate of only 9.3% (*n* = 56) for nausea and 4.7% (*n* = 29) for vomiting in the survey. There are children in whom PONV was documented in the recovery room, but they did not report it when interviewed, as well as children who indicated when interviewed that they had vomited or felt nauseous, but this did not appear in the standardized documentation. This finding is in line with Frank et al.’s study (35) who reported that only 42% of the patients experiencing PONV in the recovery room were detected by the nursing staff. This number further decreased to 29% on the ward.

In contrast in adult medicine, where nausea is easier to determine given the higher verbal ability of patients to specify their complaints, children are much less able to name complaints in concrete terms (36). Starting with school age, children can verbally report nausea as such (37, 38), an important part of patient-related outcomes as Noll et al. stated (39). The low rates of nausea and vomiting in our survey could be explained by some children's inability to accurately communicate their discomfort. They “do not think it is bad,” “probably it will go away by itself” or “that it is a part of the symptoms of an operation.” Nausea and vomiting can also be part of children's everyday life and are not very stressful for them. Unfortunately, we missed to ask the question: “How disruptive was PONV for you?” This item should be taken into consideration in further pediatric interviews regarding this topic.

Another reason for the discrepancy we found could be the timing of the event, and how well can younger children in particular remember the event if the interview took place some time after the event? Certainly, more emphasis must be placed on educating children regarding the treatability of nausea and/or vomiting in order to identify treatment gaps due to lack of coverage.

On the first and second postoperative day, the occurrence of PONV with 3.8% (*n* = 12) and 3.6% (*n* = 7) frequency, respectively, no longer played a major role. Many of the previous studies on the issue of POV/PONV in children evaluated the impact of predictors mostly in the post-anesthetic care unit (40, 41) and within 24 h following the administration of general anesthesia (6, 9, 26, 42–50). Data up to 48 h are rare (51), particularly in pediatric inpatients and after general surgery.

Ondansetron is consistently used postoperatively as an antiemetic agent both in the recovery room and on the ward, owing to its good evidence for prophylaxis and therapy in pediatric patients (3, 52, 53). There is a discrepancy between the great number of trials on the prevention of PONV and a much lesser number on its treatment. Reasons could include the fact that therapeutic trials are logistically more difficult to perform, and manufacturers may have no commercial interest in treating established emesis, assuming that all patients will receive the antiemetic drug rather than just those who need it (54).

### 4.1. Predictors of PONV

#### 4.1.1. Influence of the type of surgery on PONV

In our analysis of multivariable independent predictors of PONV, the type of surgery showed the highest OR in patients under study (OR 5.9, 95% CI: 1.8–19.2), followed by abdominal surgery (OR 3.1, 95% CI: 0.9–11.1) and urogenital tract surgery (OR 1.9, 95% CI: 0.7–5.4). Inguinal surgery was used as the reference category.

Abdominal procedures were the most affected surgical group with a prevalence rate of 52.2% (12 out of 23), followed by bone surgery (46 out of 189) with a prevalence rate of 24.3% similar to the prevalence of procedural investigations (23.8%, 10 out of 42). We found the lowest PONV prevalence after inguinal surgery (seven out of 48) at 14.6%. Balga et al. (17) demonstrated a prevalence rate of 42.9% for nausea and 19.60% for vomiting after appendectomy, and the prevalence rates of PONV following inguinal hernia repair and orchidopexy and penile surgery were 34%–50% and 37%–49%, respectively. Following inguinal surgery, Wang and Kain (55) found nausea in 65% of children in the recovery room and found vomiting in 41% of children. However, the authors noted that caudal anesthesia was not part of the study protocol, so they exclusively treated pain with intravenous opioids. This may have led to higher opioid consumption and less optimal pain control, leading to an increased prevalence of PONV (55). As previously mentioned, this study did not investigate surgeries associated with a high prevalence of PONV (strabismus surgery, adenotonsillectomy, otoplasty) (3, 6, 7, 12). What surprised us was that procedural investigations (cystoscopy *n* = 16; bronchoscopy *n* = 1; rectoscopy *n* = 7; colonoscopy *n* = 6; gastroscopy *n* = 26) were a multivariate statistically significant predictor of PONV in our sample. We performed these procedures in the operating theater at our facility and assumed that they are minimal and of short duration, so we generally did not emphasize PONV prophylaxis. Gastroscopies were the largest group of investigations. They are performed to investigate abdominal problems such as nausea/vomiting or stomach pain. The high PONV incidence may be attributed to these reasons, which warrant further investigation. However, we should respect the underlying disease that led to the examination, particularly if abdominal complaints are present.

#### 4.1.2. Perioperative administration of opioids- pain values

A statistically significant univariable predictor of PONV was the intraoperative administration of piritramide, fentanyl, and remifentanil and the administration of opioids, particularly piritramide, in the recovery room and on the ward. The findings from the multivariable analysis of independent predictors yielded consistent results, indicating that the intraoperative amount of fentanyl [2.00 (0.00–8.57) µ/kg body weight; OR 1.4, 95% CI: 1.1–1.8] and the perioperative use of piritramide (intraoperative OR 2.6. 95% CI: 1.5–4.4) in the recovery room (OR 3.0, 95% CI: 1.9–4.7) and on the ward (OR 4.5, 95% CI: 1.7–11.6) were important. The fact that the use of opioid analgesics during the perioperative period for the treatment and/or prevention of pain is a major contributing factor in children who are at risk of developing PONV has been demonstrated in several studies (9, 56–60). Bourdaud et al. (12) and Apfel et al. (61) showed that opioid application during induction of anesthesia is not by itself an independent risk factor for POV, but the administration becomes an important influence on the topic when opioids were reapplied during surgery or in the postoperative period. So “multiple opioid dose” becomes an independent risk factor of POV in the VPOP-score created by Bourdaud et al. (12), in addition to age (>3 and ≤13), duration of anesthesia (>45 min), surgery at risk (tympanoplasty, tonsillectomy, and strabismus surgery), and predisposition to POV (personal history of POV/motion sickness/familial history of POV). Therefore, it is not surprising that the Society for Pediatric Anesthesia (62) respects the high evidence and recommends, where possible, minimal use of opioids in children during the perioperative period to minimize the baseline risk for PONV. Kovac (9) explained how opioids stimulate various receptors [in the central nervous system (CNS), vestibular system, and peripherally in the gastrointestinal tract] causing nausea and vomiting. Opioid-induced nausea and vomiting (OINV) can occur for as long as 3–4 days post-surgery, with a prevalence rate of approximately 40% for nausea and of 15%–25% for vomiting (33, 63). According to the study conducted by Dinges et al. (64), there was no statistically significantly difference in the risk for PONV among different opioid compounds compared with morphine except for a lower prevalence with fentanyl and a higher prevalence with buprenorphine. After surgery, opioid use is closely associated with pain levels. We observed clinically relevant pain according to our institutional standards (FPS-r ≥4) in 27.8% (*n* = 174) of the patients and opioid use in 34% (*n* = 213) of the patients in the recovery room. On the day of surgery, 12.8% (*n* = 80) of the patients reported a pain level ≥4 on the ward. Only 25 children (4%) received an opioid. These opioid uses were a statistically significant predictor for PONV (recovery room OR 3.0, 95% CI 1.9–4.7; ward OR 4.5, 95% CI 1.7–11.6). In our survey, 64 patients (11.4%) answered that they “would have liked to receive more pain medication.” The question now arises whether the painkillers were given too sparingly or perhaps too late despite sufficient prescription. In order to optimize this, better training is needed for all professional groups involved in pain management as well as for parents and children. On the first postoperative day, 23.5% (*n* = 74) of the children still had pain scores of ≥4, probably because patients resumed normal activities and the effect of intraoperative pain management had worn off. It should be noted that 47.1% (*n* = 295) of our children never had pain during hospitalization (FPS-r = 0).

#### 4.1.3. Non-opioids/co-analgesics

With the aim of reducing opioids, we performed regional anesthesia in 44.6% (*n* = 279) of the patients, used non-opioids in 82.1% (*n* = 82.1) and co-analgesics in 31.5% (*n* = 197) of the children. Randomized controlled trials, systematic reviews, and meta-analyses have shown that multimodal opioid-sparing methods combined with the use of non-opioids (65) and co-analgesics (66, 67), local anesthetics, and regional anesthesia (when indicated) have dose-limiting opioid-sparing effects in the perioperative period (3, 68–70). They decrease the risk of any opioid-related adverse events (52, 71, 72). Therefore, it surprised us to find out that the intraoperative use of diclofenac was a statistically significant predictor of PONV in the multivariable analysis [prevalence rate of 28.9% (*n* = 59); *p* = .002; Ref. 2.02 (1.31–3.12)]. Diclofenac is safe in children older than 6 months of age, as any other NSAID (73, 74). The pharmacokinetic and pharmacodynamic properties of diclofenac and nociceptor modulation necessitate their administration in advance of the anticipated time of analgesic requirement, pre-emptively (75). The rationale behind this approach is to reach therapeutic blood levels of the NSAID before the surgical trauma induces the production of various prostaglandins (76). NSAIDs inhibit prostaglandin synthesis but do not attenuate the response to prostaglandins once they have been formed (76). NSAIDs act primarily by reversing inflammation-induced peripheral sensitization and are therefore effective in situations where this mechanism predominates (e.g., acute injuries, sports injuries, toothache). They are less effective in treating pain due to structural nerve damage (“neuropathic pain”), or in mitigating the persistent circuit changes in the transition to chronic pain (77). The literature shows that NSAIDs can lead to fewer cases of PONV (65, 78, 79) and are effective in treating postoperative pain (80). Why the use of diclofenac was a statistically significant predictor of PONV in our multivariable analysis, we can only guess after reviewing the literature. The individual factors of the patient may play an important role. We appreciated that the response to any NSAID may vary substantially from patient to patient (81). Heterogeneous mechanisms underlying the pain sensation, comorbidities, concomitant medications, and prior pain experience all contribute to inter-individual variability among those with the same diagnosis (82). The biology of the inflammatory process, the disposition of the drug (e.g., half-life, plasma concentration, concentration in inflamed tissue, penetration into the central nervous system), the duration of drug action, and possibly the degree of selectivity for inhibition of COX-2 appear to have an influence (82–84). Moreover, we have to consider that one of the side effects that can occur with diclofenac is gastrointestinal in nature, so nausea and vomiting are a possible side effect after administration. In the recovery room, the use of co-analgesics (particularly clonidine) was a statistically significant univariable predictor for PONV, also a very surprising result. Clonidine is an α2-agonist with sedative, analgesic, and antiemetic properties. In severe pain, the additional use of clonidine could reduce the dose of opioid treatment and thereby positively influence the side effects (85–87). Several studies demonstrated that the use of fentanyl but not clonidine was associated to postoperative nausea or vomiting (88–90). Goyal et al. (91) showed in their systemic review and meta-analysis that clonidine was just as effective as morphine when used as adjuvant to local anesthetic for caudal block, and had a more desirable side effect profile, particularly with respect to postoperative nausea and vomiting. In addition, the use of clonidine as a premedication was effective in reducing postoperative vomiting in pediatric patients undergoing ophthalmic surgery and provided more reduction in postoperative pain when compared with placebo (92–94).

### 4.2. Further important factors

#### 4.2.1. Introduction and maintenance of anesthesia

Looking at our forms of anesthesia, it may be statistically significant that mainly propofol was used for both induction (99.4%) and maintenance (98.1%). The antiemetic properties of propofol administered are well- documented (3, 38, 95). The main benefit of propofol is the absence of any pro-emetic activity (in contrast to the inhalational agents or ketamine). Two systematic reviews and meta-analyses (38, 40) determined that TIVA was as effective as a single antiemetic prophylactic intervention to prevent POV in pediatric patients. PONV was less frequent after general anesthesia with TIVA compared with the use of an inhalation agent alone (95). In a review of 84 studies of propofol, involving more than 6,000 patients, Tramèr et al. (41) stated that the best results in controlling nausea and vomiting were observed when propofol was used for both induction and maintenance of anesthesia. The exact mechanism of the antiemetic effect of propofol remains uncertain. Propofol may produce its antiemetic effect by depleting the area postrema of serotonin as well as by a direct gamma-aminobutyric acid-mediated inhibition (96). In addition, an attenuating effect on the cortical/subcortical afferents, a non-specific effect on the 5HT3-receptor and a reduction in the release of serotonin in the central nervous system are discussed (97). Although we performed our general anesthesia nearly in total with propofol, our PONV prevalence rate was 21.9%. So using propofol for induction and maintenance might not be enough to improve the PONV outcome.

#### 4.2.2. History of PONV/motion sickness: antiemetic application

A history of PONV was detected preoperatively in 5.3% (*n* = 33) and motion sickness in 0.6% (*n* = 4) of the patients. Only about half of them (17 out of 37) received an intraoperative prophylactic antiemetic, although a positive history was an indication for medical intraoperative prophylaxis following our guidelines and institutional standard operating procedures (3, 32, 45). An antiemetic was administered overall intraoperatively in 21.2% (*n* = 133) of the children. The most commonly administered antiemetic was dexamethasone (15.5%; *n* = 97), followed by ondansetron (8.5%; *n* = 53) and droperidol (2.1%; *n* = 13).

The same principles for PONV prophylaxis apply to children from 3 years of age as to adults (3, 6, 98). As recommended in the guidelines, pharmacological prophylaxis to protect against POV/PONV should be given in a timely manner in addition to avoiding provoking factors (3, 98, 99). Intraoperative steroids in combination with 5-HT3 receptor antagonists have the best evidence in children (1, 3, 52, 106–107). As shown by Syed et al., the introduction of specific antiemetic strategies as part of intraoperative management can reduce PONV rates to as low as 5% (108). The Cochrane review by Carlisle and Stevenson (109) analyzed 737 studies with 103,237 patients (21,632 were children and 76,842 were adults). This review determined that eight antiemetics (ondansetron, dolasetron, granisetron, tropisetron, dexamethasone, droperidol, cyclizine, and metoclopramide) reliably prevented nausea or vomiting after surgery. Interestingly, they did not find evidence that one drug was better than another. Age, sex, type of surgery, or time of drug administration did not change the effect of the drug. When drugs were given together, their effects were additive. Side effects overall were considered to be minor (109). Raval and Heiss (110) demonstrated that the central point of an enhanced recovery protocol is a decrease in complication rates due to adequate intraoperative fluid hydration, opioid-sparing analgesia, and prophylactic antiemetic administration.

It was important for us to investigate the clinical routine. The results were a real surprise for us, because we expected that antiemetic prophylaxis would be higher. Taking in account the common guidelines on this topic (3) that the administration of a PONV prophylaxis systematically to all patients above a certain age limit might be best adapted to the clinical reality, antiemetic prophylaxis was inappropriate. As anesthetists, we can influence both the intraoperative management and the procedures in the recovery room. Here, the prevalence rate of PONV was only 1.9% (12 out of 626 patients), so that anesthesiologists did not see any indication for increased emetic prophylaxis. Reasons for this may include taking fluids too quickly, feeding too soon after a surgical procedure, mobilizing too quickly on the ward, or the result of increased intraoperative and postoperative opioid use in the recovery room.

We have to be aware that the care of our patients does not end with their transfer back to the ward. Our results have shown us the importance of outcome-based therapy. Each of the pediatric anesthetists knew the guidelines, but there is a gap between the research findings and their application in clinical practice to improve outcomes and quality of care (111).

#### 4.2.3. Gender and age

Gender was not a risk factor for PONV in our dataset. In terms of age, we found the lowest prevalence of PONV in the youngest children (4 ≤ 6 years, 17.1%, *n* = 12), a prevalence rate of 21.1% (*n* = 48) in the 13 ≤ 18 years age group on the day of surgery, but age was neither a multivariable nor univariable statistically significant predictor of PONV. Khalil et al. (112) found a prevalence of 27% and 28% in children aged 1–12 and 13–24 months, respectively. Studies of children under 14 years of age found a sharp increase in PONV at 3 years of age (113), with an increase of 0.2%–0.8% per year up to puberty (7). Gold et al. (114) estimated an average PONV prevalence rate of 40% in children aged 3 years and older. Frequency slowly drops after puberty, sharing the same rate with adults (65). Only children over 4 years of age are included, so we cannot say anything about PONV prevalence in younger children.

#### 4.2.4. Duration of surgery

In the present study, the duration of surgery in minutes [OR 1.004 (95% CI: 1.000–1.008)] and the duration of anesthesia [OR 1.004 (95% CI: 1.001–1.007)] were a univariable but not multivariable predictors for PONV on the day of surgery. Operations lasting over 30 min and anesthesia period over 45 min have been accepted as risk factors in scoring PONV risks in children as Eberhart et al. (5) and Bourdaud et al. (12) demonstrated. POV prevalence rate can increase from 34% to 48% (8, 115). The reason might be a longer exposure to emetogenic stimuli (3, 116).

#### 4.2.5. Dry mouth, pain outside the surgical area, and desire for more analgesics

Children who reported that they would have liked to receive more pain medication had a higher PONV prevalence. We also observed this when children reported dry mouth or when they had “pain outside the surgical area” (Supplementary Material Table S2). On the one hand, we knew that adequate fluid therapy is a proven measure to reduce the baseline risk of PONV (3) so we paid attention to this. On the other hand, dry mouth is also a known side effect of antiemetics (117). At the time of data collection, children were allowed to drink up to 2 h before induction of anesthesia during elective procedures. However, it could be that the children did not get anything to drink for a longer time because they did not want it or their parents did not give them anything for safety reasons. Therefore, there might have been a deficit that could not be compensated by our intraoperative fluid intake.

The strengths of the present study are the extensive number of collected medical variables and the sufficient large sample size, allowing us to assure an adequate power to detect statistical significance. The present data refer to the patients’ own views of relevant points that could influence the occurrence of PONV. Furthermore, another strength of this study is the use of outcome parameters representing everyday clinical practice, rather than relying on artificial study setting.

The present study has, however, some limitations. The representativeness of our results is limited to the surgeries observed, since the surgical procedures with a high risk of PONV (ear, nose, and eye surgeries) were performed in other departments at the time of evaluation and could not be included in this study. Furthermore, this study is a single-center study, and due to the differences in technology and medication in different hospitals, the results of this study may be limited. As with any survey study, our results and conclusions are also limited by response bias. There are discrepancies between the number of self-reported and documented PONV episodes. Despite the fact that we have tried to include all available information (documentation, medication, survey), it is possible that individual PONV cases may have been overlooked. Furthermore, the PONV prevalence on the first and the second postoperative days was recorded, but it was not possible to investigate predictors for PONV on these days, due to the very low prevalence.

## 5. Conclusion

The main risk factors for PONV include administration of opioids intraoperatively, during the recovery room stay and at the ward, the use of non-opioids (diclofenac) intraoperatively, and the type of surgery performed. Real-life data demonstrated that in clinical praxis, there is a gap between the adherence to established guidelines and the use of antiemetic prophylaxis in surgeries that are generally not associated with a high prevalence of PONV. Further efforts are needed to improve the existing procedures and thus improve the outcome.

## Data Availability

The raw data supporting the conclusions of this article will be made available by the authors, without undue reservation.
