# Supplementary material for: Is PONV still a problem in pediatric surgery: a prospective study of what children tell us
Source: Front Pediatr. 2023 Oct 30;11:1241304. doi: 10.3389/fped.2023.1241304 (PMC10642485; doi:10.3389/fped.2023.1241304)
Supplement: Supplementary file 1 [file Table1.docx]

Supplementary Material

Is PONV still a problem in pediatric surgery: a retrospective study of what children tell us

**Brigitte Messerer^1^†, Marko Stijic^2,3^** **†, Andreas Sandner-Kiesling^1^, Johanna M. Brillinger^2^, Jasmin Helm^2^, Horst J. Lindner^1^, Jacqueline Scheer^2^, Christof Stefan Strohmeier^2^, Alexander Avian^2*^**

^1^ Department of Anesthesiology and Intensive Care Medicine, Medical University Graz, Austria

^2^ Institute for Medical Informatics, Statistics and Documentation, Medical University Graz, Austria

^3^ University Clinic for Neurology, Clinical Department for Neurogeriatrics, Medical University Graz

† These authors have contributed equally to this work and share first authorship.

*** Correspondence:**Corresponding Author

Avian Alexander

Institute for Medical Informatics, Statistics and Documentation, Medical University Graz, Austria Auenbruggerplatz 2

8036 Graz, Austria

Tel.: +43 – 316 – 385 – 17873

Fax.: +43 – 316 – 385 – 13590

alexander.avian@medunigraz.at

# Supplementary Data

*Supplementary Table 1.* Further patient characteristics.

| **Characteristic** | **n (%)**  **median (min-max)** |
| --- | --- |
| Only nausea |  |
| Day of surgery (ward)  No  Yes | 599 (95.7)  27 (4.3) |
| 1^st^ postoperative day  No  Yes | 310 (98.4)  5 (1.6) |
| 2^nd^ postoperative day  No  Yes | 190 (99.0)  2 (1.0) |
| Only Vomiting |  |
| Day of surgery (ward)  No  Yes | 538 (86.1)  87 (13.9) |
| 1^st^ postoperative day  No  Yes | 308 (97.8)  7 (2.2) |
| 2^nd^ postoperative day  No  Yes | 187 (97.4)  5 (2.6) |
| PONV |  |
| Day of surgery (ward)  No  Yes | 525 (83.9)  101 (16.1) |
| 1^st^ postoperative day  No  Yes | 303 (96.2)  12 (3.8) |
| 2^nd^ postoperative day  No  Yes | 185 (96.4)  7 (3.6) |
| Intraoperative |  |
| Airway management  Intubation  LAMA  spontan | 99 (15.8)  512 (81.8)  15 (2.4) |
| Antiemetic |  |
| Antiemetic - intraoperative  No  Yes | 493 (78.8)  133 (21.2) |
| Dexamethason**-** intraoperative  No  Yes | 529 (84.5)  97 (15.5) |
| Dexamethason **-** intraoperative mg/ kgBW | .00 (.00-1.09) |
| Ponveridol**-** intraoperative  No  Yes | 613 (97.9)  13 (2.1) |
| Ondansetron **-** intraoperative  No  Yes | 567 (91.5)  53 (8.5) |
| Ondansetron **-** intraoperative mg/ kgBW | .00 (.00-0.13) |
| Antiemetic Day of surgery (ward)  No  Yes | 548 (87.5)  78 (12.5) |
| Antiemetic 1^st^ postoperative day  No  Yes | 310 (98.4)  5 (1.6) |
| Antiemetic 2^nd^ postoperative day  No  Yes | 190 (99.0)  2 (1.0) |
| PCA |  |
| Day of surgery  No  Yes | 576 (92.0)  50 (8.0) |
| 1^st^ postoperative day  No  Yes | 267 (84.8)  48 (15.2) |
| 2^nd^ postoperative day  No  Yes | 157 (81.8)  35 (18.2) |
| Length of hospital stay | 1 (0 – 13) |
| **Day of Surgery (recovery room + ward)** |  |
| PONV  No  Yes | 489 (78.1)  137 (21.9) |
| **Survey questions:** |  |
| Were you nauseous?  No  Yes | 543 (90.7)  56 (9.3) |
| Have you vomited?  No  Yes | 583 (95.3)  29 (4.7) |

*Supplementary Table 2*: Univariable logistic regression results (Outcome: PONV on day of surgery (including recovery room)

| **Characteristic** | **n (%)**  **median (min-max)** | | | **PONV Prevalence** | | **p-value** | | **OR (95%CI)** | |
| --- | --- | --- | --- | --- | --- | --- | --- | --- | --- |
| Sex  Male  Female | 405 (64.7)  221 (35.3) | | | 19.5% (n=79)  26.2% (n=58) | | .052 | | Ref.  0.68 (0.46 – 1.00) | |
| Age | 11.08 (4.04-17.99) | | |  | | .807 | | 0.99 (0.95 – 1-04) | |
| Age groups  4 - <6  6 - <10  10 - <13  13 - <18 | 70 (11.2)  200 (31.9)  128 (20.4)  228 (36.4) | | | 17.1% (n=12)  24.0% (n=48)  22.7% (n=29)  21.1% (n=48) | | .666  Ref.  .237  .361  .476 | | Ref.  1.53 (0.76 – 3.08)  1.42 (0.67 – 2.99)  1.29 (0.64 – 2.59) | |
| Body mass index z-score | .32 (-4.92-5.09) | | |  | | .780 | | 1.02 (0.89 – 1.17) | |
| Body mass index z-score  < -1  - 1 to 1  > 1 | 103 (16.5)  319 (51.0)  202 (32.3) | | | 17.5% (n=18)  22.6% (n=72)  23.3% (n=47) | | .480  Ref.  .274  .244 | | Ref.  1.38 (0.78 – 2.44)  1.43 (0.78 – 2.62) | |
| ASA-score  1  2  3 | 455 (72.7)  165 (26.4)  6 (1.0) | | | 21.5% (n=98)  22.4% (n=37)  n = 2 | |  | |  | |
| ASA-score  1  ≥ 2 | 455 (72.7)  172 (27.5) | | | 21.5% (n = 98)  23.3% (n = 40) | | .732 | | Ref.  1.08 (0.71 – 1.64) | |
| Duration of surgery in minutes | 31 (1-348) | | |  | | **.040** | | 1.004 (1.000 – 1.008) | |
| Duration of surgery  < 30 minutes  ≥ 30 minutes | 303 (48.4)  323 (51.6) | | | 20.1% (n=61)  23.5% (n=76) | | .305 | | Ref  1.22 (0.83 – 1.79) | |
| Duration of anaesthesia in minutes | 68 (9-434) | | |  | | **.011** | | 1.004 (1.001 – 1.007) | |
| Duration of anaesthesia  ≤ 45 minutes? Group 1.0  > 45 minutes? Group 2.0 | 130 (20.8)  496 (79.2) | | | 17.7% (n=23)  23.0% (n=114) | | .195 | | Ref.  1.39 (0.85 – 2.28) | |
| Duration of anaesthesia - Duration of surgery, | 36 (4 -140) | | |  | | **.004** | | 1.02 (1.00 – 1.03) | |
| History of PONV/ motion sickness  no  PONV and/or motion sickness | 589 (94.1)  37 (5.9) | | | 21.9% (n=129)  21.6% (n=8) | | n < 20 | | Ref.  0.98 (0.44 – 2.20) | |
| Type of surgery  Inguinal surgery  Plastic surgery  Bones  Knee, shoulder, hip, joints  Minor surgery  Urogenital tract  Abdominal procedures  Investigations | 48 (7.7)  27 (4.3)  189 (30.2)  55 (8.8)  129 (20.6)  113 (18.1)  23 (3.7)  42 (6.7) | | | 14.6% (n=7)  18.5% (n=5)  24.3% (n=46)  23.6% (n=13)  20.9% (n=27)  15.0% (n=17)  52.2% (n=12)  23.8% (n=10) | | **.024**  Ref.  **.007**  .057  .915  .478  .152  .507  .942 | | Ref.  3.39 (1.40 – 8.20)  0.55 (0.30 – 1.02)  0.96 (0.48 – 1.95)  0.82 (0.48 – 1.41)  0.53 (0.22 – 1.26)  0.71 (0.25 – 1.97)  0.97 (0.44 – 2.13) | |
| Pre-medication |  | |  | | |  | |  | |
| Midazolam  no  yes | 14 (2.2)  612 (97.8) | | 21.4% (n=3)  21.9% (n=134) | | | .967 | | 1.03 (0.28 – 3.74) | |
| Catapresan  no  yes | 624 (99.7)  2 (0.3) | | 22.0% (n=137)  n=0 | | | n < 20 | |  | |
| Type of anaesthesia  General anaesthesia GA  GA+ Regional anaesthesia  (perph.RA, PDA, SS-Caudal, infiltration, PWB)  Analgosedation  Spinal anaesthesia | 332 (53.0)  278 (44.4)  15 (2.4)  1 (0.2) | | 25.3% (n=84)  18.7% (n=52)  6.7% (n=1)  n=0 | | | n < 20 | |  | |
| Type of anaesthesia  General anaesthesia GA  GA+ Regional anaesthesia  (perph.RA, PDA, SS-Caudal, infiltration, PWB) | 332 (53.0)  278 (44.4) | | 25.3% (n=84)  18.7% (n=52) | | | .052 | | Ref.  0.68 (0.46 – 1.00) | |
| Induction  Propofol  vs other | 622 (99.4)  4 (0.6) | | 21.9% (n=136)  n=1 | | | n < 20 | |  | |
| Maintenance of anaesthesia  Propofol  vs other | 614 (98.1)  12 (1.9) | | 21.8% (n=134)  25.0% (n=3) | | | n < 20 | |  | |
| **Opioids (intraoperative)**  no  yes | 11 (1.8)  615 (98.2) | | 9.1% (n=11)  22.1% (n=136) | | | n < 20 | |  | |
| Piritramide  no  yes | 284 (45.4)  342 (54.6) | | 14.1% (n=40)  28.4% (n=97) | | | **<.001** | | Ref.  2.42 (1.61 – 3.64) | |
| Piritramide µg/ kgBW | 79.6(0.0 – 374.3) | |  | | | **<.001** | | 1.010 (1.006 – 1.013) | |
| Fentanyl  no  yes | 60 (9.2)  566 (90.4) | | 20.0% (n=2)  22.1% (n=125) | | | .711 | | Ref.  1.13 (0.58 – 2.20) | |
| Fentanyl µg/ kgBW | 2.00(.00-8.57) | |  | | | **.009** | | 1.33 (1.07 – 1.64) | |
| Remifentanil  no  yes | 375 (59.9)  251 (40.1) | | 19.2% (n=72)  25.9% (n=65) | | | **.048** | | Ref.  1.47 (1.00 – 2.15) | |
| Remifentanil µg/ kgBW | 0.00 (.00-55.43) | |  | | | **.003** | | 1.04 (1.01 – 1.06) | |
| **Non-Opioid intraoperative**  No  yes | 107 (17.1)  519 (82.1) | | 19.6% (n=21)  22.4% (n=116) | | | .535 | | Ref.  1.18 (0.70 – 1.98) | |
| Ibuprofen  No  yes | 526 (84.0)  100 (16.0) | | 23.2% (n=122)  15.0% (n=15) | | | .**062** | | Ref.  0.58 (0.33 – 1.05) | |
| Ibuprofen mg/ kgBW | .00 (.00-12.5) | |  | | | **.062** | | 0.94 (0.88 – 1.00) | |
| Diclofenac  No  yes | 422 (67.4)  204 (32.6) | | 18.5% (n=78)  28.9% (n=59) | | | **.003** | | Ref.  1.80 (1.22 – 2.65) | |
| Diclofenac mg/ kgBW | .00 (.00-2.27) | |  | | | **<.001** | | 1.80 (1.28 – 2.53) | |
| Neodolpasse  No  yes | 451 (72.0)  175 (28.0) | | 22.8% (n=103) 19.4% (n=34) | | | .355 | | Ref.  0.82 (0.53 – 1.26) | |
| Neodolpasse ml/ kgBW | .00 (.00-4.39) | |  | | | .325 | | 0.93 (0.81 – 1.07) | |
| Metamizol  No  yes | 581 (92.8)  45 (7.2) | | 21.5% (n=125) 26.7% (n=12) | | | .442 | | Ref.  1.33 (0.67 – 2.64) | |
| Metamizol mg/ kgBW | .00 (.00-15.79) | |  | | | .470 | | 1.03 (0.96 – 1.10) | |
| Intraop. application of one non-opioid  no non-opioid  one non-opioid  two non-opioid | 107 (17.1)  514 (82.1)  5 (0.8) | | 19.6% (n=21)  21.8% (n=112)  n =4 | | | n < 20 | |  | |
| **Co-analgesics intraoperative**  No  yes | 429 (68.5)  197 (31.5) | | 22.4% (n=96)  20.8% (n=41) | | | .660 | | Ref.  0.91 (0.60 – 1.38) | |
| Ketanest  No  Yes | 524 (83.7)  102 (16.3) | | 21.9% (n=115) 21.6% (n=22) | | | .933 | | Ref.  0.98 (0.58 – 1.64) | |
| Ketanest mg/ kgBW | .00 (.00-2.34) | |  | | | .758 | | 1.10 (0.59 – 2.05) | |
| Catapresan  No  Yes | 510 (81.5)  116 (18.5) | | 22.0% (n=112)  21.6% (n=25) | | | .923 | | Ref.  0.98 (0.60 – 1.59) | |
| Catapresan µg/ kgBW | .00 (.00-4.44) | |  | | | .714 | | 1.06 (0.79 – 1.41) | |
| Intraoperative fluid volume ml/ kgBW | 311.0 (0.0-7322.0) | |  | | | .239 | | 1.01 (0.99 – 1.04) | |
| **Recovery room** | |  |  | |  | |  | |  |
| Time of stay in minutes | | 104 (19-276) |  | | **<.001** | | 1.012 (1.006 – 1.018) | |  |
| Maximal pain  0-3  ≥4 | | 452 (72.2)  174 (27.8) | 16.2% (n=73) 36.8% (n=64) | | **<.001** | | Ref.  3.02 (2.03 – 4.49) | |  |
| pain value when transferred to the inpatient  0  1  2  3  4 | | 423 (67.6  128 (20.4)  52 (8.3)  21 (3.4)  2 (0.3) | 19.9% (n=84)  22.7% (n=29)  30.8% (n=16)  28.6% (n=6)  n=2 | | n < 20 | |  | |  |
| pain value when transferred to the inpatient  ≤2  3 / 4 | | 603 (96.3)  23 (3.7) | 21.4% (n=129)  34.8% (n=8) | | .134 | | Ref.  1.96 (0.81 – 4.72) | |  |
| Opioide  No  yes | | 413 (66.0)  213 (34.0) | 15.0% (n=62) 35.2% (n=75) | | **<.001** | | Ref.  3.08 (2.08 – 4.54) | |  |
| Piritramide  No  Yes | | 442 (70.6)  184 (29.4) | 16.5% (n=73) 34.8% (n=64) | | **<.001** | | Ref.  2.70 (1.82 – 4.00) | |  |
| Piritramide µg/ kgBW | | 0.0 (0.0 – 153.4) |  | | **<.001** | | 1.012 (1.008 – 1.017) | |  |
| Nalbuphin  No  Yes | | 606 (96.8)  20 (3.2) | 21.6% (n=131) 30.0% (n=6) | | .376 | | Ref.  1.55 (0.59 – 4.12) | |  |
| PCA  No  Yes | | 612 (97.8)  14 (2.2) | 21.2% (n=131) 30.0% (n=6) | | n < 20 | |  | |  |
| Non-opioid  No  Yes | | 451 (72.0)  175 (28.0) | 21.7% (n=98) 22.3% (n=39) | | .880 | | Ref.  1.03 (0.68 – 1.57) | |  |
| Metamizol  No  Yes | | 459 (73.3)  167 (26.7) | 22.2% (n=102)  21.0% (n=35) | | .735 | | Ref.  0.93 (0.60 – 1.43) | |  |
| Metamizol mg/ kgBW | | .00 (.00-20.00) |  | | .790 | | 0.99 (0.95 – 1.04) | |  |
| Neodolpasse  No  Yes | | 619 (98.9)  7 (1.1) | 21.5% (n=133)  n=4 | | n < 20 | |  | |  |
| Buscopan  No  Yes | | 624 (99.7)  2 (0.3) | 21.8% (n=136)  n=1 | | n < 20 | |  | |  |
| **Co-analgesics**  No  Yes | | 521 (83.2)  105 (16.8) | 20.2% (n=105) 30.5% (n=32) | | **.021** | | Ref.  1.74 (1.09 – 2.77) | |  |
| Catapresan  No  Yes | | 522 (83.4)  104 (16.6) | 20.3% (n=106)  29.8% (n=31) | | .**034** | | Ref.  1.67 (1.04 – 2.67) | |  |
| Catapresan µg/ kgBW | | .00 (.00-2.88) |  | | .108 | | 1.34 (0.94 – 1.90) | |  |
| Ketanest  No  Yes | | 618 (98.7)  8 (1.3) | 21.4% (n=132)  n=6 | | n < 20 | |  | |  |
| Ondansetron  No  Yes | | 614 (98.1)  12 (1.9) | 20.4% (n=125)  100.0% (n=12) | | n < 20 | |  | |  |
| Total liquid ml/ kgBW | | 5.45(.00-127.71) |  | | .573 | | 1.01 (0.98 – 1.03) | |  |
| Total amount of Piritramide Intraop. + recovery room  mg/ kgBW | | 91.8 (0.0-374.3) |  | | **<.001** | | 1.008 (1.006 – 1.011) | |  |
| **OP+Rec Room** | |  |  | |  | |  | |  |
| administration of non-opioids  total of no non-opioids  total of one non-opioids  total of ≥ two non-opioids | | 72 (11.5)  473 (75.6)  81 (12.9) | 18.1% (n=13)  21.8% (n=103)  25.9% (n=21) | | .500  Ref.  .473  .245 | | Ref.  1.26 (0.67 – 2.39)  1.59 (0.73 – 3.46) | |  |
| **On the WARD** | |  |  | |  | |  | |  |
| Piritramide  No  Yes | | 601 (96)  25 (4.0) |  | | **<.001** | | Ref.  4.17 (1.86 – 9.36) | |  |
| Pain | |  |  | |  | |  | |  |
| Day of surgery  0  1-3  4-10 | | 330 (52.8)  215 (34.4)  80 (12.8) | 18.2% (n = 60)  21.4% (n = 46)  38.8% (n = 31) | | **<.001**  Ref.  .355  **<.001** | | Ref.  1.23 (0.80 – 1.88)  2.85 (1.68 – 4.84) | |  |
| Non opioid | |  |  | |  | |  | |  |
| Day of surgery  No  Yes | | 342 (54.6)  284 (45.4) | 20.5% (n = 70)  23.6% (n = 67) | | .347 | | Ref.  1.20 (0.82 – 1.75) | |  |
| Medication on demand | |  |  | |  | |  | |  |
| Day of surgery  No  Yes | | 575 (91.9)  51 ( 8.1) | 20.2% (n=116)  41.2% (n=21) | | **<.001** | | Ref.  2.77 (1.53 – 5.02) | |  |
| **Survey questions:** | |  |  | |  | |  | |  |
| Were you thirsty?  No  Yes | | 208 (34.2)  400 (65.8) |  | | .069 | | Ref.  1.48 (0.97 – 2.26) | |  |
| Was your mouth dry?  No  Yes | | 187 (32.7)  385 (67.3) |  | | **.015** | | Ref.  1.74 (1.11 – 2.73) | |  |
| Would you have liked to receive more pain medication?  No  Yes | | 497 (88.6)  64 (11.4) |  | | **<.001** | | Ref.  2.86 (1.66 – 4.94) | |  |
| Did you have pain outside of the surgical area?  How severe was it?  0  1-3  >4 | | 499 (83.9)  55 ( 9.1)  41 (7.0) |  | | **.001**  Ref.  **.013**  **.002** | | Ref.  2.15 (1.17 – 3.95)  2.83 (1.45 – 5.52) | |  |
| Out of this group:  n=39 had abdominal pain:  0  1-3  >4 | | 21 (53.8)  9 (23.1)  9 (23.1) |  | | .166  Ref.  .402  .060 | | Ref.  2.00 (0.40 – 10.11)  5.00 (0.93 – 26.79) | |  |
